# Observations on Homœopathy

**Published:** 1884-04

**Authors:** 


### OBSERVATIONS ON HOMŒOPATHY.

There are three propositions which appertain exclusively to Homœopathy, and which constitute its essence :

1. To try the medicaments on healthy persons before administering them to the diseased.

2. To choose every medicament according to the analogy of its symptoms.

3. To administer but single medicaments, and in suitable doses.

The first of these propositions is so true and so evident, that we do not conceive how medicine could have existed so many centuries before Hahnemann without its having been proclaimed and put in practice by physicians.

He who opposes its progress does it without a better conviction, acting merely from a spirit of opposition; he does not know what he says, because he *defends nonsense*. This principle will last forever; the whole world cannot destroy it.

The second proposition is not less true, although less evident at first sight; it has been violently attacked by thousands of physicians, but there is no question here of the partisans of the ancient medical system, who, for want of experience, cannot possibly pass an impartial judgment; but physicians even, who have actually adopted this principle, have asked what we understand by analogy. Hahnemann understands by analogy the morbid symptoms carefully compared with those of the medicament to be chosen, keeping account of the characteristic and important symptoms. The medicament which reproduces exactly the accidents of a given disease answers the best, according to him, for the affection, and promises to become a true specific. We have, according to our treatment, two problems to resolve.

1. To ascertain the nature of each morbid affection, and to choose a remedy whose effects on the healthy body resemble the diseases to be cured.

Hahnemann *seems* only to reject that knowledge which we have a right to expect from every well-informed physician; but when we consider the great care he has taken in the examination of a disease, that he did not neglect the slightest circumstance, that he distinguished with an extraordinary talent the essential symptoms from those which are unessential; when we reflect, besides, that he has characterized in a masterly manner in the notes to the first two volumes of his *Materia Medica* the tendency of the action of the medicaments, we cannot doubt that he has made use of that physiological and pathological knowledge which he possessed in so eminent a degree.

How could he have discovered, without this knowledge, that *Conium maculatum* is a specific in the hypochondriasis peculiar to men of stern habits, and that *Solanum nigrum* is the very remedy for egotism? We need not mention many other cases, which prove in a most incontestable manner his talent in this respect. Those who flatter themselves that they follow him, but have not the requisite knowledge, are very much mistaken.

The instructive notes with which Hahnemann has enriched his first two volumes of the *Materia Medica* become more scarce in the subsequent ones. I am convinced it would have been better had he not discontinued them. The experiments since published by his disciples are poor in such annotations; the consequence is, that those of less experience do not know how to use them. Every one has not the faculty of making Hahnemann-like experiments with medicaments; very few can be compared with him in this respect.

Many homœopathists have declared that it is sufficient to know the *general* effect of each medicament upon the healthy body, the principal tendency of its action, without considering the slighter shades peculiar to the action of a medicine. In the practice, they limit themselves to catch the *ensemble* of each disease, without descending to the special symptoms, and they administer the remedy which seems to be analogous. In this manner they prescribe for an atony of the stomach, with perverse secretions, *Acidum sulphuricum;* for a sur-excitation of the nerves of the stomach, with atony of its muscular fibres, *Nux vomica;* for cramps of the stomach, with a disposition to constipation and a predominant lymphatic system, *Conium maculatum;* for constipation, accompanied and kept up by general weakness, with a less active circulation, *Ferrum metallicum:* what the medicaments want in quality is replaced by quantity. In this manner we fall back to the generalization of the old school, and this extreme direction is as fatal to the progress of art as the opposite method, which consists in covering mechanically the symptoms, without appreciation of their physiological dependence.

We shall reach our aim much safer by keeping the middle point (*juste milieu*); most homœopaths follow that course, and become thus more perfect in their diagnosis and in their knowledge of the materia medica.

The third proposition of Hahnemann, to administer but single medicaments, and in suitable doses, is as evident as the first. What reasonable opposition can the old school make to the new on this point? Can they defend their mixture of drugs—an absurdity which most of them despise in their hearts? They will renounce it when they understand their medicaments better. Their whole materia medica is but the fabrication of ignorance, and the iron hand by which they intend to oppose all movement of liberty in therapeutics will in time prove beneficial to the only right mode of making experiments with medicaments.

It is true that the administering two medicaments at the same time has been recommended, but every judicious mind considers this an absurdity. We might as well mix three or four remedies; this would be a backward step, and open the door to the gross empiricism of the old school.—*Gross.*

